# Storage and time course effects on the quality of oil extracted from *Phyllanthus amarus* Schumach and *Annona muricata* Linn and their antidiabetic potentials

**DOI:** 10.5114/bta.2024.135641

**Published:** 2024-03-29

**Authors:** Oluwasayo E. Ogunjinmi, Vincent O. Oriyomi, Richard A. Olaogun, Amidat T. Gbadegesin

**Affiliations:** 1 Department of Industrial Chemistry, First Technical University, Ibadan, Nigeria; 2 Department of Biochemistry and Forensic Science First Technical University, Ibadan, Nigeria

**Keywords:** *Phyllanthus amarus*, *Annona muricata*, phytochemicals, diabetes, rancidity, gout

## Abstract

With the advent of modern technology, advancements in processing and storage techniques, and increasing medical knowledge, people are becoming aware of deterioration in the quality of medicinal products due to storage methods and time. In most cases, herbal products are not consumed immediately after production; as such, improper storage can result in physical, chemical, and microbiological changes. The study evaluated the effect of storage methods and time on the quality of oil extracted from *Phyllanthus amarus* Schumach and *Annona muricata* Linn and assessed their antidiabetic and antioxidative effects. Plants were air-dried, pulverized, and then subjected to Soxhlet extraction in petroleum ether. The oil was evaluated for phytochemical constituents and the effects of time and storage methods on its physicochemical properties. Characterization of the oil was done by spectroscopic techniques. Oils from both plants contained tannins, flavonoids, alkaloids, steroids, glycosides, terpenoids, phlobotannins, resins, reducing sugar, phenols, and saponins in different proportions. The oil from *A. muricata* had higher phenolic (3.11 ± 0.31 mg GAE/g), flavonoid (11.82 ± 0.08 mg QUE/g), alkaloid (16.37 ± 0.56 mg APE/g), and tannin (7.13 ± 0.47 mg CE/g) contents than the oil from *P. amarus*, which had 0.54 ± 0.08 mg GAE/g, 7.83 ± 0.13 mg QUE/g, 9.87 ± 0.15 mg APE, and 3.16 ± 0.12 mg CE/g for total phenolic, flavonoids, alkaloids, and tannins, respectively. Initial acid, iodine, peroxide, and saponification values recorded for *P. amarus* were 5.63 ± 0.82 mg KOH/g, 97.17 ±0.53 Wijis, 9.31 ± 0.15 mEq/kg, and 116.11 ± 0.74 mg KOH/g, respectively, significantly different from those of *A. muricata* , which had values of 1.17 ± 0.08 mg KOH, 76.23 ± 0.03 Wijis, 6.75 ± 0.47 mEq/kg, and 193.31 ± 0.52 mg KOH/g, respectively. FT-IR characterization of the oils revealed the presence of carboxylic acid, alkyl, alkene, alkane, haloalkane, aldehyde, aromatic amine, α-unsaturated and β-unsaturated esters, and phenol functional groups. *P. amarus* oil inhibited α-amylase (IC_50_ 0.17 ± 0.03 mg/ml), α-glucosidase (IC_50_ 0.64 ± 0.03 mg/ml), and xanthine oxidase (0.70 ± 0.01 mg/ml) to a greater extent than *A. muricata* oil, with IC_50_ values of 0.43 ± 0.05 mg/ml (α-amylase), 2.25 ± 0.31 mg/ml (α-glucosidase), and 0.78 ± 0.07 mg/ml (xanthine oxidase). This study showed that oils from the tested plants have low rancidity with a moderate shelf life. The extracts contained essential phytoconstituents that significantly inhibited α-glucosidase and xanthine oxidase. These effects of the oil indicate their potential to prevent diabetes, gout, and oxidative stress. Consequently, the supply of *P. amarus* and *A. muricata* in homemade diets is strongly encouraged for healthy living.

## Introduction

Nature provides numerous valuable resources to humanity, including plants with therapeutic uses. Many orally formulated synthetic substances used for treatment come with significant side effects and are sometimes ineffective in chronic conditions (Saberi and Ataee, 2021). Builders and Philip (2019) report that 80% of Earth’s inhabitants rely on traditional medicine for primary healthcare needs, often in the form of active principles derived from plants. Ancient civilizations depended on these remedies for treating various illnesses and infectious diseases (Jamshidi-Kia et al., 2018).

Phytocomponents, such as phenolics, flavonoids, tannins, anthocyanidins, and various plant or herbal extracts, have been reported as radical scavengers and inhibitors of oxidative stress (Kaushik et al., 2021). It is reasonable to assert that the health benefits of a plant depend on its chemical constituents, shelf-life, and storage period, which produce specific physiological actions in the human body (Singh, 2015). Consequently, there is a growing demand for natural products with high pharmacological potential and minimal or no side effects.

With the advent of modern technology, advances in processing and storage techniques, and increasing medical knowledge, people are becoming aware of deterioration in the quality of medicinal products due to storage methods and time. Herbal products are often not consumed immediately after production, leading to the possibility of physical, chemical, and microbiological changes. According to Lisboa et al. (2018), the recommended storage period for medicinal plants is 1 year. The methods and duration of storage for medicinal products hold significant importance in the production chain, directly influencing the quality and quantity of active principles. Proper storage ultimately reduces metabolic activity and the potential deterioration of medicinal products. Therefore, ensuring an appropriate storage method for herbal products is crucial to enhancing the stability of their active principles over time (Lisboa et al., 2018).

*Phyllanthus amarus*, belonging to the family Euphorbiaceae, is an annual tropical plant known for its various medicinal benefits in folkloric medicine. According to reports, *P. amarus* exhibits numerous medicinal properties, including antihepatitis, antimalarial, anticarcinogenic, antiasthmatic, antiviral, antibacterial, antidiarrheal, hepatoprotective, anti-inflammatory, and antidiabetic properties (Ogunmoyole et al., 2020; Olorunnisola et al., 2021). Additionally, the use of *Annona muricata* is well-documented for addressing conditions such as cancer, fever, hemolysis, respiratory illness, malaria, gastrointestinal problems, liver, heart, and kidney affections, hypoglycemia, hypotension, and cancer (Gavamukulya et al., 2017; Badmus et al., 2022). Despite the various medicinal values of these plants, there is a dire need to address the challenges posed by the effects of storage methods and time on their nutraceutical values. This study aims to evaluate the time course and storage effects on the quality of the oil derived from these plants, along with assessing their potential as antidiabetic agents and in the treatment of gout.

## Materials and methods

### Plant collection

The aerial parts of *P. amarus* and seeds of *A. muricata* fruits were harvested within the premises of First Technical University, Ibadan, Oyo State, Nigeria, situated at latitude 7˚39′12″ and longitude 3˚91′67″ during the dry season (November/December 2022). The collection and authentication of plant samples were conducted by a taxonomist at the Department of Pure and Applied Biology, Ladoke Akintola University of Technology, Ogbomoso, Oyo State, Nigeria, with voucher number (LHB/4618) and were deposited at the University Herbarium. The plant materials were cleaned under running tap water, sundried, and later powdered using a mechanical grinder (SR-14733, Marlex, Daman and Diu, India) according to Manuwa et al. (2020). The powder obtained was stored in a desiccator until required.

### Soxhlet extraction of plant materials

The extraction of oil from plant materials was carried out according to the method of Redfern et al. (2014). At each time, sufficient plant materials were used to fill the porous cellulose thimble (approximately 15 g of the sample in a 25 × 80-mm thimble). Subsequently, 250 ml of petroleum ether was added to a round-bottom flask connected to a Soxhlet extractor, and a condenser on an isomantle was attached. The crushed plant material was loaded into the thimble, which was placed inside the Soxhlet extractor, with the side arm lagged with glass wool. The solvent was heated using the isomantle for evaporation, moving through the apparatus to the condenser. The condensate dripped into the reservoir containing the thimble. Once the solvent reached the siphon, it poured back into the flask, and the cycle repeated. The process continued for a total of 18 h. After completion, the petroleum ether was evaporated using a rotary evaporator, resulting in a small yield of extracted plant oil collected in the glass bottom flask (5 ml).

The collected samples were then placed into cleaned and dried Eppendorf tubes, divided into three portions. Two different storage methods, dark shelf storage and refrigeration (at 4˚C), were utilized. Each portion was stored either in the dark (cupboard) or at ambient light (shelf) or in the refrigerator for a duration ranging from 1 to 42 days. The physicochemical properties of the stored oil were evaluated at 2-week intervals (14 days) until the 42^nd^ day.

### Determination of the percentage of oil yield

Thirty grams (30 g) of the sample were carefully placed in the thimble, and approximately 150 ml of petroleum ether was poured into the round-bottom flask. The apparatus was then heated within the range of 40–60%, allowing for a continuous 3 h extraction using the Soxhlet apparatus. This experimental procedure was repeated for varying sample weights: 35, 40, and 50 g. Following the extraction process, the solvent was distilled, and the percentage of oil extracted was determined based on the dry weight of the sample.

### Qualitative phytochemical analyses of oil

A phytochemical test was conducted to identify the presence or absence of secondary metabolites, including alkaloids, flavonoids, glycosides, saponins, tannins, quinines, carbohydrates, resins, etc. The methods employed for these tests were in accordance with the procedures outlined by Attaullah et al. (2020).

### Quantitative phytochemical analyses

The atropin equivalent of the extracts was used to determine their total alkaloid content (TAC) according to the method of Shamsa et al. (2008). For the determination of total flavonoid content (TFC), the quercetin acid equivalent of the fractions was measured, following the method described by Sun et al. (1999). The total tannin content (TTC) of the extracts was determined by spectrophotometrically measuring the catechin equivalent of the fractions, according to the procedure by Singh et al. (2012). Furthermore, the total phenolic content (TPC) was determined using a spectrophotometric method, as described by Singleton et al. (1999), Gulcin et al. (2004). All analyses were performed in three repetitions.

### Determination of physicochemical parameters

The physicochemical parameters were measured to assess the stability of the oils was assessed through the measurement of various physicochemical parameters. The iodine values (IV) of the oil samples were determined by Wijs’ method, following the procedures outlined by Kyari (2008) and Nwakodo et al. (2018). The amount of iodine absorbed is a measure of the degree of oxidation of the oil (Pike and O’Keefe, 2017). For the determination of acid value (AV), a mixture of oil sample (10 g), diethyl ether (25 ml), ethanol (25 ml), and drops of phenolphthalein was titrated against 0.1M NaOH. The appearance of a dark pink color indicated the endpoint of the reaction, signifying the formation of free fatty acids (FFA). Saponification value (SV) was measured using the indicator method, involving the addition of a few drops of phenolphthalein to a warm sample solution before titration, in accordance with the procedures outlined by AOCS (1986). The amount of fat saponified was then calculated. Peroxide value (PV) was determined through titration with a mixture of oil sample (1 g), potassium iodide (1 g), and 20 ml solvent mixture (glacial acetic acid/chloroform, 2:1 v/v) (Kaboré et al., 2020). These parameters were measured for the different storage methods and change in time, and performed in triplicates.

### Fourier Transform Infrared (FT-IR) characterization of extracted oils

Extracts were subjected to a Fourier Transform Infrared Resonance (FT-IR) Spectrometer of universal absorbance transmittance resonance (ATR) on a Perkin-Elmer Frontier FTIR Spectrometer with serial #100858 using spectrum software version 6.1. The measurements were conducted at temperatures between 25 and 27˚C, recording spectra from 4500 to 400/cm with a spectral resolution of 4/cm. Peak frequencies were compared to the reference literature (Stuart, 2005) and the IR correlation chart (Okpo and Otaraku, 2020) to evaluate the functional groups present in oils extracted from *P. amarus* and *A. muricata*.

### Enzyme inhibition studies of extracted oils

The alpha (α)-glucosidase inhibitory study followed the method described by Li et al. (2005). The extract’s ability to inhibit α-glucosidase activity was determined by measuring the release of yellow-colored paranitrophenol from pNPG, monitored at 405 nm, with acarbose serving as a reference inhibitor. In vitro, α-amylase inhibitory assays were carried out according to Wickramaratne et al. (2016). The generation of reducing sugar was quantified by the reduction of 3,5-dinitrosalicylic acid to 3-amino-5-nitrosalicylic acid, with acarbose used as a standard. The reaction mixture was monitored at 540 nm using a spectrophotometer. For the measurement of xanthine oxidase inhibition activity, the quantification of uric acid formed by the catalysis of xanthine by xanthine oxidase was conducted using UV Spectrophotometry. Xanthine oxidase activity was determined following the modified method of Olanrewaju et al. (2018) with xanthine as the substrate. The oil was prepared in a stock solution (1 mg/ml) in phosphate buffer and used for the xanthine oxidase inhibition assay. Allopurinol, prepared in a buffer, served as the reference compound (positive control). A triplicate analysis of the inhibitory potential of the oil on α-amylase and α-glucosidase was performed.

### Statistical analysis

Statistical analysis was carried out using SPSS version 16.0 software (SPSS Inc., Chicago, IL, USA). Values were expressed as mean ± standard error of the mean. Comparisons were performed using a one-way analysis of variance followed by Tukey’s test (as the posthoc test). Differences at *P* < 0.05 were taken as significant. The IC_50_ values were calculated using GraphPad PRISM version 6 software (La Jolla, CA, USA).

## Results and discussion

### Yield of oil extracted from Phyllanthus amarus and Annona muricata

The yield of oils extracted from dried aerial parts of *P. amarus* and seeds of *A. muricata* were obtained in percentages of 0.43 ± 0.08 (v/w) and 1.32 ± 0.03 (v/w), respectively.

### Preliminary screening of the phytochemical constituents of P. amarus and A. muricata oil

The results of the phytochemical analysis of the oil of *P. amarus* and *A. muricata* are presented in Table 1. The oils exhibited a trace to the average presence of reducing sugar, phlobotannins, tannins, saponins, and an appreciable presence of alkaloids, flavonoids, phenols, and terpenoids. Notably, the plants tested negative for glycosides. According to Fabio (2017), these phytocompounds have high economic value due to their significance in combating diseases and improving overall health. In the extract from *A. muricata*, all tested phytochemicals were present, except for reducing sugar, phlobatannins, and glycosides (Table 1). This finding contrasts with the results reported by Naik and Sellappan (2019), who observed the absence of phenolics and flavonoids in the root, leaf, bark, and rind of *A. muricata* extracted with ether by maceration. Algandaby (2015) and Azwinda (2015) highlighted that the presence or absence of phytochemicals depends on various factors, including the test method, solvent, particle size, moisture content, solvent-to-sample ratio, pH, temperature, length of extraction, extraction method, plant part, and origin/environment.

**Table 1:** Qualitative phytoconstituents in petroleum ether extracted oils from *Phyllanthus amarus* and *Annona muricata*

S/No	Phytochemical	*Phyllanthus amarus*	*Annona muricata*
1	Tannins	++	+
2	Glycosides	–	–
3	Resins	+++	+++
4	Saponins	++	+
5	Phlobotannins	+	–
6	Flavonoids	+++	+
7	Steroids	+++	+++
8	Phenolics	+++	++
9	Reducing sugars	+	–
10	Alkaloids	+++	+
11	Terpenoids	+++	+++

(–) – absent, (+) – trace amount, (++) – average amount, (+++) – appreciable amount

This investigation reports an appreciable level of steroids (+++), resins (+++), and terpenoids (+++) in the extract obtained from *A. muricata* seeds. Consistent with this, Agu and Okolie (2017) previously reported *A. muricata* as a rich source of terpenoids, esters of aliphatic acids, volatile oils, derivatives of long-chain fatty acids (C32 or C34), and high levels of nonpolar essential oils in different plant parts.

Table 2 presents the total phenolic (TPC), tannin (TTC), flavonoid (TFC), and alkaloid (TAC) contents of petroleum ether-extracted oils from *P. amarus* and *A. muricata*. These values were estimated from gallic, catechin, quercetin, and atropine standard curves, respectively. The amounts of these phytochemicals in *A. muricata* were significantly higher than in *P. amarus*. Specifically, *A. muricata* exhibited higher TPC, TTC, TFC, and TAC (3.11 ± 0.31 mg GAE/g, 7.13 ± 0.47 mg CE/g, 11.82 ± 0.08 mg QUE/g, and 16.37 ± 0.56 mg APE/g oil) compared to *P. amarus* (0.54 ± 0.08 mg GAE/g, 3.16 ± 0.12 mg CE/g, 7.83 ± 0.13 mg QUE/g, and 9.87 ± 0.15 mg APE/g). Reports of higher yields of TPC, TTC, TFC, and more than the value reported in this study using the Soxhlet extraction technique are many (Aman et al., 2019; Osman et al., 2019). Yields of phytochemicals obtained by using Soxhlet extraction were also comparable to those by supercritical fluid-aided extraction methods (Bourgou et al., 2017). A high content of these phytochemicals was reported in the aqueous and ethanol extracts of both plants and a range of 50.51 ± 3.21 to 560.21 ± 6.22 mg/100 g for the TPC while the total flavonoid contents ranged from 85.65 ± 7.63 to 275.45 ± 10.01 mg/100 g (Gavamukulya et al., 2014; Adefegha et al., 2015; Nguyen et al., 2020; Yakubu et al., 2021).

**Table 2:** Quantitative phytoconstituents in petroleum ether extracted oils from *Phyllanthus amarus* and *Annona muricata*

S/No	Component	*P. amarus* (mean ± SEM)	*A. muricata* (mean ± SEM)
1	total phenolic content [mg GAE/g]	0.54 ± 0.08 ^b^	3.11 ± 0.31 ^a^
2	total flavonoid content [mg QUE/g]	7.83 ± 0.13 ^a^	11.82 ± 0.08 ^a^
3	total alkaloid content [mg APE/g]	9.87 ± 0.15 ^a^	16.37 ± 0.56 ^b^
4	total tannin content [mg CE/g]	3.16 ± 0.12 ^b^	7.13 ± 0.47 ^a^

GAE – gallic acid equivalent, CE – catechin equivalent, QUE – quercetin equivalent, APE – atropine equivalent; each value is presented as mean ± SEM; means with the same superscript letters (a–d) in a row are significantly different (*P* > 0.05, Tukey’s test), while those with different superscript letters are significantly different (*P* < 0.05, Tukey’s test)

### Physicochemical properties of extracted oils from P. amarus and A. muricata based on storage time and methods

The presence of free fatty acids causes rancidity in oils (Emmanuel et al., 2017; Aguebor-Ogie et al., 2020). Oils with high levels of free fatty acids are more susceptible to oxidative aging and tend to become rancid more quickly (Adegbola et al., 2018; Liu et al., 2019). The free fatty acids are a major fat fuel in the body, however, their elevated levels in the blood raise the risk of cardiovascular disease due to insulin resistance (which can lead to diabetes) and elevated blood pressure (Adegbola et al., 2017, 2018).

Essential fatty acids, such as linoleic acid (18 : 2; 9, 12), arachidonic acid (20 : 4; 5, 8, 11, 14), and linolenic acid (18 : 3; 9, 12, 15), are crucial forms of fatty acids required by the body in dietary fats and oils (Jafari et al., 2022). These essential fatty acids play essential roles in various bodily functions, including the transport of cholestasis, formation of lipoproteins, prevention of fatty liver, synthesis of important compounds like eicosanoids, and maintenance of membrane function and structure (Joerg and Ludger, 2021). Deficiency in essential fatty acids can result in conditions such as phrynoderma/toad skin, characterized by horny eruptions on the posterior and lateral parts of limbs, back, buttocks, hair loss, and poor wound healing (Leah et al., 2015).

To assess the quality of oils, common indices include iodine, acid, peroxide, and saponification values (Adegbola et al., 2018; Aguebor-Ogie et al., 2020).

The AV is often utilized as a general indicator of the condition and edibility of an oil. This is because an increase in acid value is accompanied by the development of objectionable odors and flavors (Nwokem et al., 2010). The acid value is used as an indicator of the edibility of oil. The lower the acid value of oil, the fewer the fatty acid it contains which makes it less exposed to the phenomenon of rancidity (Nielson, 1994). In the present study (Table 3), the initial AV of *P. amarus* (5.63 ± 0.82) was higher than that of *A. muricata* (1.17 ± 0.08) and that of the RCS. Similarly, both oils showed an increase in AV after 42 days of storage (Table 4), possibly due to the high free fatty acid content in *P. amarus* oil and the potential absorption of moisture during storage (Chabiri et al., 2009). The increase in AV of oil stored on the shelf (ambient light condition) compared to those stored in the dark and in the refrigerator may be attributed to the photodecomposition of the oil contents (Table 5). According to Nwokodo et al. (2019), iodine value (IV) is the quantity of iodine absorbed by 1 g of the oil to saturate the sigma bond. A lower iodine value means that the oils are stable and hence have a greater shelf life. A high IV is used to determine the degree of FFA/unsaturation, presence of high moisture content (Akubor and Ogu, 2012), high impurities, high rancidity/autoxidation, and high oxidation. The occurrence of higher IV in *P. amarus* (Tables 3 and 4) is indicative of its degree of unsaturation. From the result of IV presented in Table 5, it can be concluded that the oils from tested plants are most stable when kept away from light or stored under 4˚C. The assessment of PV is a measure of deterioration (rancidity) in unsaturated fats and oils, among other available methods (Dobarganes and Velasco, 2002). PV gives a measure of the degree to which an oil has undergone primary oxidation (Gbalade and Soremekun, 1997). A high peroxide value leads to the elevation of peroxide and free radicals, which are capable of causing massive inflammation, aging, cancer, atherosclerosis, and damage to tissue. The PV determined that oils from *P. amarus* and *A. muricata* (Tables 3–5) fall within the recommended standard limit, indicating their nutritive and pharmacological potentials.

**Table 3:** Initial physicochemical properties of oils extracted from *Phyllanthus amarus* and *Annona muricata*

Parameter	*P. amarus*	*A. muricata*	RCS
Acid value [mg KOH/g]	5.63 ± 0.82	1.17 ± 0.08	0.60
Iodine value [Wijis]	97.17 ± 0.53	76.23 ± 0.03	75–94
Peroxide value [mEq/kg]	9.31 ± 0.15	6.75 ± 0.47	10 max
Saponification value [mg KOH/g]	116.11 ± 0.74	193.31 ± 0.52	184–196

mEq/kg – milliequivalent/kg, RCS – recommended codex standard

**Table 4:** Physicochemical properties of *Phyllanthus amarus* and *Annona muricata* oil based on storage time

Plant	Parameter	After day 7	After day 14	After day 21	After day 28	After day 35	After day 42
*P. amarus*	acid value	5.71 ± 0.02	5.77 ± 0.07	5.79 ± 0.10	6.31 ± 0.33	7.56 ± 0.13	7.81 ± 0.48
	iodine value	98.31 ± 0.63	98.79 ± 0.11	99.08 ± 0.07	99.16 ± 0.73	101.71 ± 0.06	103.86 ± 0.13
	peroxide value	9.49 ± 0.63	8.61 ± 0.81	9.51 ± 1.08	9.58 ± 1.16	9.72 ± 0.61	9.77 ± 0.88
	saponification value	114.87 ± 0.36	113.10 ± 0.19	112.80 ± 0.38	110.11 ± 0.81	109.13 ± 0.11	107.34 ± 0.91
*A. muricata*	acid value	1.16 ± 0.66	1.16 ± 0.12	1.21 ± 0.34	1.22 ± 0.44	1.23 ± 0.27	1.25 ± 0.18
	iodine value	77.34 ± 0.62	77.18 ± 0.35	78.73 ± 0.67	81.26 ± 1.05	81.49 ± 0.74	81.88 ± 0.05
	peroxide value	6.79 ± 1.04	6.87 ± 0.18	6.91 ± 0.97	6.98 ± 0.44	7.12 ± 0.71	7.23 ± 0.84
	saponification value	193.31 ± 0.52	194.18 ± 0.06	196.87 ± 0.07	198.73 ± 0.84	198.97 ± 0.11	199.71 ± 0.54

**Table 5:** Physicochemical properties of the oils from *Phyllanthus amarus* and *Annona muricata* based on storage methods

Source	Parameter	Shelf			Dark			Refrigerator		
		2 weeks	4 weeks	6 weeks	2 weeks	4 weeks	6 weeks	2 weeks	4 weeks	6 weeks
*P. amarus*	acid value	5.77 ± 0.07	6.31 ± 0.33	7.81 ± 0.48	5.11 ± 0.12	5.21 ± 0.07	5.68 ± 0.38	3.62 ± 0.01	3.82 ± 0.04	3.47 ± 0.06
	iodine value	98.79 ± 0.11	99.16 ± 0.73	103.86 ± 0.13	95.51 ± 0.13	96.27 ± 0.08	97.11 ± 0.01	93.08 ± 0.14	93.89 ± 0.07	94.66 ± 0.03
	peroxide value	8.61 ± 0.81	9.58 ± 1.16	9.77 ± 0.88	7.83 ± 0.12	7.91 ± 0.18	8.08 ± 0.33	6.48 ± 0.03	6.51 ± 0.37	6.61 ± 0.04
	saponification value	113.10 ± 0.19	110.11 ± 0.81	107.34 ± 0.91	129.11 ± 0.06	125.08 ± 0.10	123.06 ± 0.04	138.58 ± 0.07	136.91 ± 0.24	132.57 ± 0.33
*A. muricata*	acid value	1.16 ± 0.12	1.22 ± 0.44	1.25 ± 0.18	1.06 ± 0.01	1.09 ± 0.03	1.14 ± 0.22	0.98 ± 0.02	1.00 ± 0.01	1.08 ± 0.02
	iodine value	77.18 ± 0.35	81.26 ± 1.05	81.88 ± 0.05	73.31 ± 0.10	74.11 ± 0.34	75.39 ± 0.21	69.76 ± 0.35	71.18 ± 0.31	73.83 ± 0.42
	peroxide value	6.87 ± 0.18	6.98 ± 0.44	7.23 ± 0.84	5.88 ± 0.34	6.17 ± 0.16	6.28 ± 0.31	4.87 ± 0.36	5.93 ± 0.30	6.18 ± 0.14
	saponification value	194.18 ± 0.06	198.73 ± 0.84	199.71 ± 0.54	203.64 ± 0.11	200.92 ± 0.62	198.01 ± 0.11	223.73 ± 0.06	218.37 ± 0.31	214.61 ± 0.08

The SV reflects the molecular weights of FFA (triglycerides) in oils. Higher SV is attributed to a predominantly high proportion of shorter carbon chain lengths of fatty acids (Kirk and Sawyer, 1999). The saponification value is inversely proportional to the average molecular weight or length of fatty acids (Muhammad et al., 2011; Riris and Silalahi, 2017). Therefore, the shorter the average chain length (C_4_–C_12_) the higher the saponification value. Saponification value gives information on the solubility of oil in water (Akinola et al., 2010). The SV results in this study revealed that oil stored in the refrigerator had the highest value compared to those kept on the shelf and in the dark cupboard (Tables 3–5), suggesting that the oils are best stored in the refrigerator and away from light. Additionally, the SV of both *P. amarus* and *A. muricata* decreased with the time of storage, implying an increase in fatty acid content over time. Overall, the results suggest that the oil of *A. muricata* has a longer shelf life than the oil of *P. amarus*.

### Fourier Transform Infrared Spectroscopic characterization of oils from A. muricata and P. amarus

**Fig. 1:**
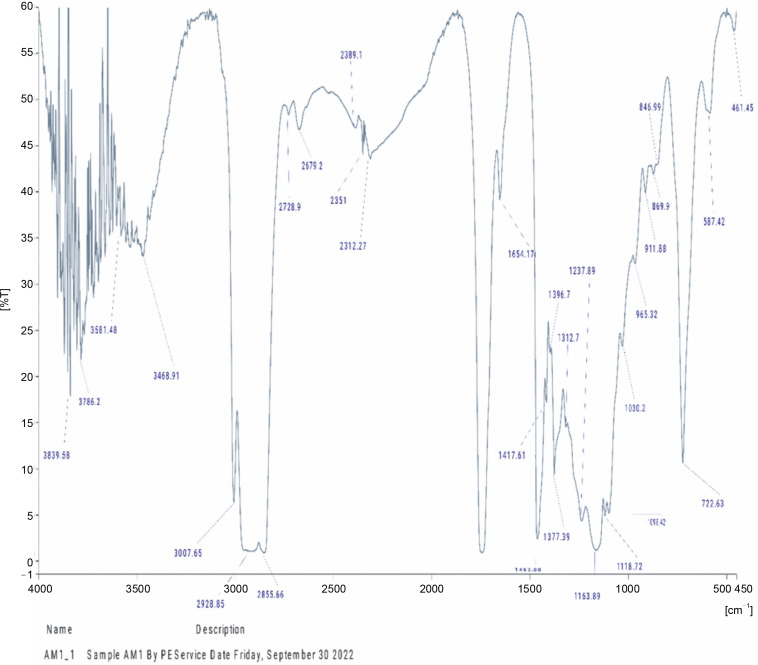
FTIR spectra of petroleum ether oil from *Annona muricata*

**Fig. 2:**
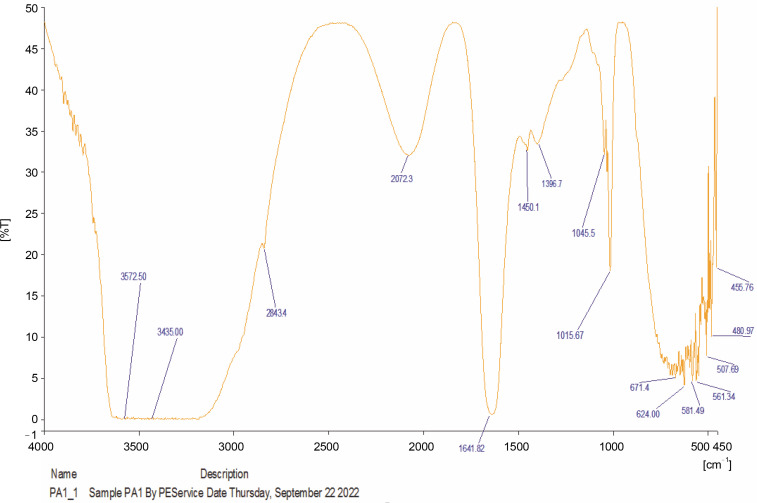
FTIR spectra of petroleum ether oil from *Phyllanthus amarus*

The compounds identified in the tested oils, as determined by FT-IR analyses, are summarized in Table 6, and the corresponding spectra peaks are shown in Figures 1 and 2. The essential oil of *A. muricata* exhibited sixteen (16) distinct peaks (Fig. 1), while *P. amarus* oil displayed thirty (30) distinct peaks (Fig. 2). In *A. muricata* oil, the most prominent groups included alcohols (3839.58 and 3468.91/cm), carboxylic acids (3007.65 and 1654.17/cm), and alkyl halides (722/cm). Conversely, in *P. amarus*, the predominant groups were carboxylic acids with a peak value of 3572.50/cm, isothiocyanate (2072.30/cm), alkanes (1641.82/cm), and alcohols/esters (1015.67/cm). The spectra also indicated the presence of minor groups such as alkenes, haloalkanes, aryl disulfides, polysulfides, aromatic, and heterocyclic compounds.

**Table 6:** Functional groups present in oils of *Phyllanthus amarus* and *Annona muricata*

	*Annona muricata*			*Phyllanthus amarus*		
S/No	PV (cm^−1^)	Bond	Group	PV (cm^−1^)	Bond	Group
1	3839.58^m^	N–H, O–H, C-H	alcohols	3572.50^b^	O–H; C–H; N-H	alcohols
2	3786.20^s^	O–H	alcohols	3433.00^b^	O–H stretch,=N–H	alcohol; heterocyclic amines
3	3581.48^s,b^	O–H, H–bonded	phenols, alcohols	2843.40^m^	C–H stretching	alkane
4	3468.91^s,b^	O–H, H–bonded	alcohols	2072.30^s^	N=C=S	isothiocyanate
5	3007.65^m^	O–H, =C–H	CA, alkenes	1641.82^m^	–C=C–	alkanes
6	2928.85^m^	C–H	alkenes	1450.10^m^	C–C	aromatics
7	2855.66^m^	C–H	alkenes	1396.70^s^	C–F	fluoro compounds
8	2728.90^s^	H–C=O:C–H	aldehydes	1045.50^s^	C–O	alcohols, ester, ether, CA
9	2679.20^s,b^	O–H bonded	CAs	1015.67^s^	C–O	alcohols, ester, ether, CA
10	2389.10^w^	C/C	alkyne disubstituted	671.40^b, s^	–C=C–; C–H	alkynes
11	2351.0^s,b^	N=C=O	isocyanate	624.00^b,s^	–C/C–H:C–H bend	alkynes
12	2312.27^w^	C/N	nitrile	581.49^m^	C–Br	alkyl halides
13	1719.83^s^	C=O	α,β-unsaturated ester	561.34^m^	C–Br	alkyl halides
14	1654.17^m^	–C=C–; C=O	alkenes, CAs	507.69^m^	C–I	organohalogens
15	1463.88^m^	C–H bend	alkanes	480.97	S–S stretch	arly disulfide, polysulfides
16	1417.61^m^	C–C stretch (in-ring)	aromatics	455.76	S–S stretch	arly disulfide, polysulfides
17	1396.70^m^	O–H bending	CAs			
18	1377.39^m^	O–H bending	alcohol, CAs			
19	1312.70^s^	C–N stretch	aromatic amines			
20	1237.89^s^	C–O	alcohols, ester, ether, CAs			
21	1163.89^s^	C–O	alcohols, ester, ether, CAs			
22	1118.72^m^	C–H wag -CH_2_X	alkyl halides			
23	1030.20^s^	C–O	alcohols, ester, ether, CAs			
24	965.320^s^	=C–H bend	alkenes			
25	911.88^b^	O–H bend	CAs			
26	869.90^s, b^	N–H Wag	1E, 2E amines			
27	846.99^s^	N–H Wag, C-H “oop"	aromatics			
28	722.63^m^	C–Cl stretch, C–H	alkyl halides, aromatics			
29	587.42^b,s^	–C/C–H:C–H bend	alkynes			
30	461.45	S–S Stretch	aryl disulfides			

PV – peak value, CA – carboxylic acid, m – medium, s – strong, b – broad, w – weak

The abundance of multiple vibrations, including C=O, N–H, O–H, and C=C, in the spectra suggest the presence of beneficial secondary metabolites such as flavonoids, terpenes, and polyphenols (Mazurek et al., 2013; Akwu et al., 2019).

### Inhibitory activities of extracted oils from P. amarus and A. muricata on α-amylase, α-glucosidase, and xanthine oxidase

The *in vitro* inhibitory effects of *P. amarus* and *A. muricata* oil extracts on the activity of α-amylase, α-glucosidase, and xanthine oxidase are presented in Table 7. *P. amarus* oil exhibited the highest inhibitory effect towards α-amylase, α-glucosidase, and xanthine oxidase with IC_50_ values of 0.17 ± 0.03, 0.64 ± 0.03, and 0.70 ± 0.01, respectively, compared to *A. muricata* oil with IC_50_ values of 0.43 ± 0.05, 2.25 ± 0.31, and 0.78 ± 0.07 for α-amylase, α-glucosidase, and xanthine oxidase, respectively. The inhibition of these enzyme activities by the extracted oils and standard drugs (acarbose and allopurinol) was found to be dose-dependent (Table 7). The IC_50_ values of *P. amarus* oil on the activities of α-amylase (0.17 ± 0.03 mg/ml), α-glucosidase (0.64 ± 0.03 mg/ml), and xanthine oxidase (0.70 ± 0.01 mg/ml) were significantly (*P* < 0.05) lower than those of *A. muricata*.

**Table 7:** *In vitro* inhibitory effects of *Phyllanthus amarus* and *Annona muricata* extracted oils on α-amylase, α-glucosidase, and xanthine oxidase (XO) activities

Oil	Concentration [mg/ml]	Inhibition ± SEM [%]			IC_50_ [mg/ml] ± SEM		
		α-amylase	α-glucosidase	XO	α-amylase	α-glucosidase	XO
*P. amarus*	1	59.38 ± 0.47	54.68 ± 0.73	61.62 ± 1.61	0.17 ±0.03	0.64 ± 0.03	0.70 ± 0.01
	0.5	58.69 ± 0.30	46.92 ± 0.49	49.02 ± 0.82			
	0.25	56.79 ± 1.64	45.04 ± 0.61	35.25 ± 0.20			
	0.125	52.65 ± 0.18	43.73 ± 0.62	27.05 ± 0.76			
	0.0625	48.77 ± 0.73	40.34 ± 0.08	25.57 ± 0.57			
	0.03125	39.20 ± 0.93	38.43 ± 0.47	8.57 ± 0.04			
	0.015625	29.37 ± 1.64	31.80 ± 0.85	1.68 ± 0.66			
*A. muricata*	1	51.10 ± 1.01	43.96 ± 0.58	55.31 ± 1.08	0.43 ± 0.05	2.25 ± 0.31	0.78 ± 0.07
	0.5	48.25 ± 1.10	44.18 ± 1.29	47.89 ± 0.71			
	0.25	44.37 ± 1.28	43.64 ± 1.52	32.13 ± 0.39			
	0.125	41.27 ± 1.02	41.80 ± 0.62	23.59 ± 0.47			
	0.0625	34. 02 ± 1.28	39.52 ± 0.15	13.58 ± 0.83			
	0.03125	26.78 ± 1.74	37.89 ± 0.39	7.688 ± 0.43			
	0.015625	16.69 ± 0.73	37.10 ± 0.37	1.96 ± 0.39			

To the best of our knowledge, this is the first report on the enzyme inhibitory properties of these oils on α-amylase, α-glucosidase, and xanthine oxidase under these experimental conditions. Efforts to optimize conditions for isolating components eliciting these inhibitory effects in the oils are ongoing in our laboratory. The claim is that botanical pharmacological agents that are excellent inhibitors of α-amylase and α-glucosidase have fewer side effects and are less expensive than synthetic pharmacotherapeutics like acarbose, glibenclamides, metformin, febuxostat, miglitol, and others has increased the interest in investigating them (Gong et al. 2020).

Pharmacotherapeutic suppression of α-amylase and α-glucosidase activity has been proposed as advantageous for diabetes mellitus patients with impaired insulinotropic response, particularly when combined with other oral hypoglycemic medications (Yosief et al., 2018). The ability of the inhibitors of α-amylase and α-glucosidase to reduce weight gain or improve weight loss in nondiabetics has also been reported. The antidiabetic effects of oils from the two plants may be associated with the presence of phytoconstituents, especially flavonoids, alkaloids, terpenoids, steroids, and saponins, which were present in appreciable amounts.

Determination of xanthine oxidase activity is relevant for diagnostic purposes (Kostic et al., 2015). Allopurinol, along with other known xanthine oxidase inhibitors like oxypurinol and febuxostat, has been widely used for the treatment of diabetes, gout, and hyperuricemia (Bredemeier et al., 2018; Cicero et al., 2021). Nonreversible inhibitors of xanthine oxidase are potent antioxidant agents of vascular oxidative stressors important for treating oxidative stress-induced conditions like diabetes, nephropathy, hyperuricemia, and others (Takahashi et al., 2018; Rullo et al., 2023). The inhibitory effects of *P. amarus* and *A. muricata* oils suggest their potential to effectively manage these conditions, possibly due to the presence of secondary metabolites, including flavonoids, phenolics, alkaloids, and tannins, known for their scavenging properties against free radicals.

## Conclusion

*P. amarus* and *A. muricata* are medicinal plants widely utilized for managing various conditions in folkloric and alternative medicine. This study investigates the effects of storage methods and duration on the physicochemical and pharmacological activities of the oil extracted from these medicinal plants. Optimal preservation of the pharmacologically crucial phytoconstituents is achieved through refrigeration and protection from light. These oils present viable alternatives to major edible oils like palm, soybeans, coconut, and groundnut.

## Supplementary Material

Storage and time course effects on the quality of oil extracted from *Phyllanthus amarus* Schumach and *Annona muricata* Linn and their antidiabetic potentials
