# Supplementary material for: Storage and time course effects on the quality of oil extracted from Phyllanthus amarus Schumach and Annona muricata Linn and their antidiabetic potentials
Source: BioTechnologia (Pozn). 2024 Mar 29;105(1):41–53. doi: 10.5114/bta.2024.135641 (PMC11020154; doi:10.5114/bta.2024.135641)
Supplement: Storage and time course effects on the quality of oil extracted from Phyllanthus amarus Schumach and Annona muricata Linn and their antidiabetic potentials [file BTA-105-1-52456-s001.pdf]

BioTechnologia vol. 105 (1) • 2024
Journal of Biotechnology, Computational Biology and Bionanotechnology SUPPLEMENTARY MATERIALS
https://doi.org/10.5114/bta.2024.135641

CC BY 4.0

Storage and time course effects on the quality of oil extracted from *Phyllanthus amarus* Schumach and *Annona muricata* Linn and their antidiabetic potentials

Oluwasayo E. Ogunjinmi [1]*, Vincent O. Oriyomi [2], Richard A. Olaogun [1], Amidat T. Gbadegesin [1]

[1] Department of Industrial Chemistry, First Technical University, Ibadan, Nigeria

[2] Department of Biochemistry and Forensic Science First Technical University, Ibadan, Nigeria

Received: 2 May 2023; revised: 1 December 2023; accepted: 22 December 2023

Abstract

With the advent of modern technology, advancements in processing and storage techniques, and increasing medical knowledge, people are becoming aware of deterioration in the quality of medicinal products due to storage methods and time. In most cases, herbal products are not consumed immediately after production; as such, improper storage can result in physical, chemical, and microbiological changes. The study evaluated the effect of storage methods and time on the quality of oil extracted from *Phyllanthus amarus* Schumach and *Annona muricata* Linn and assessed their antidiabetic and antioxidative effects. Plants were air-dried, pulverized, and then subjected to Soxhlet extraction in petroleum ether. The oil was evaluated for phytochemical constituents and the effects of time and storage methods on its physicochemical properties. Characterization of the oil was done by spectroscopic techniques. Oils from both plants contained tannins, flavonoids, alkaloids, steroids, glycosides, terpenoids, phlobotannins, resins, reducing sugar, phenols, and saponins in different proportions. The oil from *A. muricata* had higher phenolic (3.11 ± 0.31 mgGAE/g), flavonoid (11.82 ± 0.08 mgQUE/g), alkaloid (16.37 ± ± 0.56 mgAPE/g), and tannin (7.13 ± 0.47 mgCE/g) contents than the oil from *P. amarus*, which had 0.54 ± ± 0.08 mgGAE/g, 7.83 ± 0.13 mgQUE/g, 9.87 ± 0.15 mgAPE, and 3.16 ± 0.12 mgCE/g for total phenolic, flavonoids, alkaloids, and tannins, respectively. Initial acid, iodine, peroxide, and saponification values recorded for *P. amarus* were 5.63 ± 0.82 mgKOH/g, 97.17 ±0.53 Wijis, 9.31 ± 0.15 mEq/kg, and 116.11 ± 0.74 mgKOH/g, respectively, significantly different from those of *A. muricata*, which had values of 1.17 ± 0.08 mgKOH, 76.23 ± ± 0.03 Wijis, 6.75 ± 0.47 mEq/kg, and 193.31 ± 0.52 mgKOH/g, respectively. FT-IR characterization of the oils revealed the presence of carboxylic acid, alkyl, alkene, alkane, haloalkane, aldehyde, aromatic amine, α-unsaturated and β-unsaturated esters, and phenol functional groups. *P. amarus* oil inhibited α-amylase (IC_{50} 0.17 ± ± 0.03 mg/ml), α-glucosidase (IC_{50} 0.64 ± 0.03 mg/ml), and xanthine oxidase (0.70 ± 0.01 mg/ml) to a greater extent than *A. muricata* oil, with IC_{50} values of 0.43 ± 0.05 mg/ml (α-amylase), 2.25 ± 0.31 mg/ml (α-glucosidase), and 0.78 ± 0.07 mg/ml (xanthine oxidase). This study showed that oils from the tested plants have low rancidity with a moderate shelf life. The extracts contained essential phytoconstituents that significantly inhibited α-glucosidase and xanthine oxidase. These effects of the oil indicate their potential to prevent diabetes, gout, and oxidative stress. Consequently, the supply of *P. amarus* and *A. muricata* in homemade diets is strongly encouraged for healthy living.

Key words: *Phyllanthus amarus*, *Annona muricata*, phytochemicals, diabetes, rancidity, gout

* Corresponding author: Department of Industrial Chemistry, First Technical University, Ibadan, Nigeria;
e-mail: oluwasayo.ogunjinmi@tech-u.edu.ng

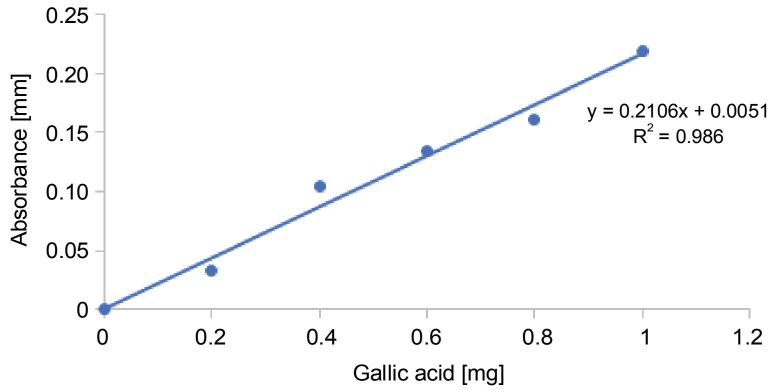

Appendix I. Calibration curve for total phenol content

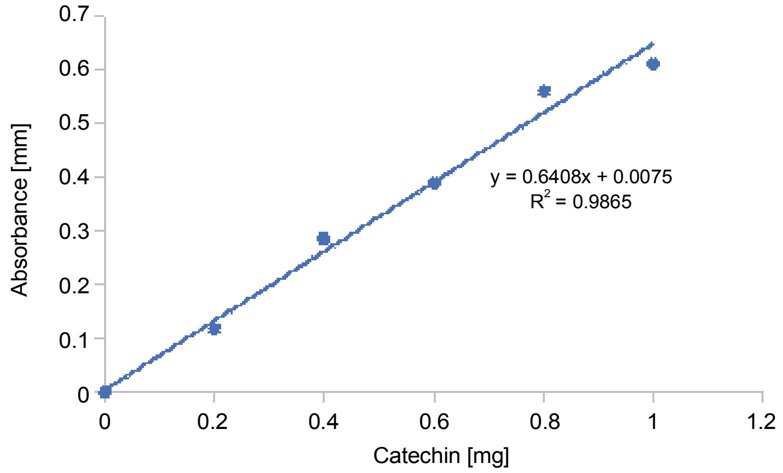

Appendix II. Calibration curve for total tannin content

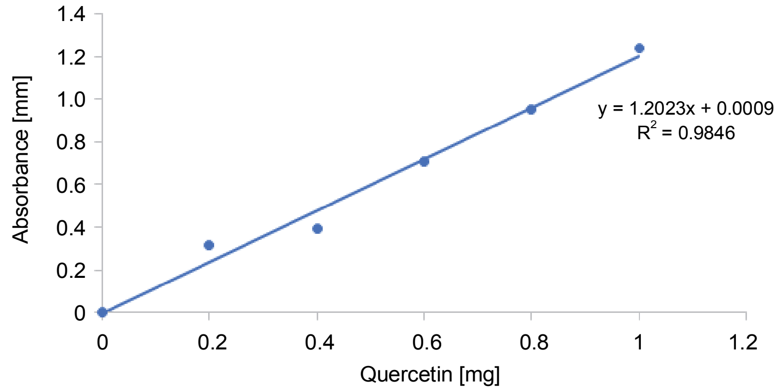

Appendix III. Calibration curve for total flavonoid content

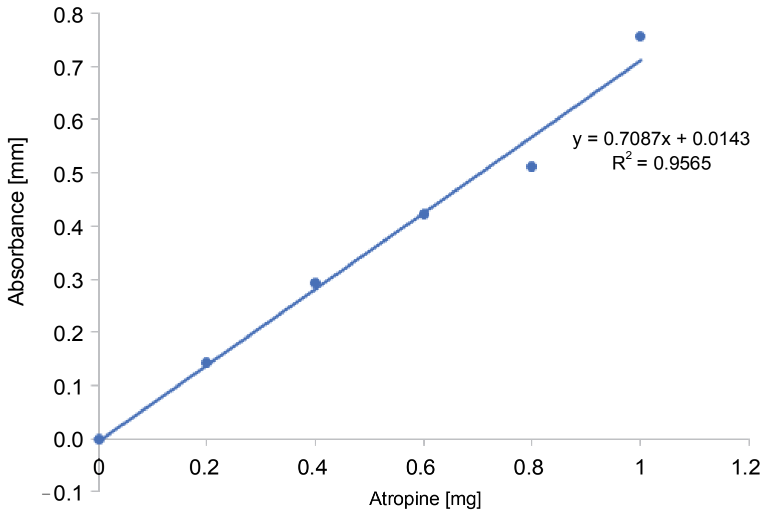

Appendix IV. Calibration curve for total alkaloid content
